# Unveiling the synergistic effect of precursor stoichiometry and interfacial reactions for perovskite light-emitting diodes

**DOI:** 10.1038/s41467-019-10612-3

**Published:** 2019-06-27

**Authors:** Zhongcheng Yuan, Yanfeng Miao, Zhangjun Hu, Weidong Xu, Chaoyang Kuang, Kang Pan, Pinlei Liu, Jingya Lai, Baoquan Sun, Jianpu Wang, Sai Bai, Feng Gao

**Affiliations:** 1 Department of Physics, Chemistry and Biology (IFM), Linköping University, Linköping, SE-58183 Sweden; 2 Key Laboratory of Flexible Electronics (KLOFE) and Institute of Advanced Materials (IAM), Jiangsu National Synergetic Innovation Center for Advanced Materials (SICAM), Nanjing Tech University (Nanjing Tech), Nanjing, 211816 China; 3 Jiangsu Key Laboratory for Carbon-Based Functional Materials & Devices, Institute of Functional Nano & Soft Materials (FUNSOM), Joint International Research Laboratory of Carbon-Based Functional Materials and Devices, Soochow University, Suzhou, 215123 China

**Keywords:** Chemistry, Energy science and technology, Engineering, Materials science, Optics and photonics

## Abstract

Metal halide perovskites are emerging as promising semiconductors for cost-effective and high-performance light-emitting diodes (LEDs). Previous investigations have focused on the optimisation of the emissive perovskite layer, for example, through quantum confinement to enhance the radiative recombination or through defect passivation to decrease non-radiative recombination. However, an in-depth understanding of how the buried charge transport layers affect the perovskite crystallisation, though of critical importance, is currently missing for perovskite LEDs. Here, we reveal synergistic effect of precursor stoichiometry and interfacial reactions for perovskite LEDs, and establish useful guidelines for rational device optimization. We reveal that efficient deprotonation of the undesirable organic cations by a metal oxide interlayer with a high isoelectric point is critical to promote the transition of intermediate phases to highly emissive perovskite films. Combining our findings with effective defect passivation of the active layer, we achieve high-efficiency perovskite LEDs with a maximum external quantum efficiency of 19.6%.

## Abstract (web summary)

Metal halide perovskite light-emitting diodes (PeLEDs) attract lots of attention but the role of charge transport layers is less known. Here, Yuan *et al*. explore the effects of precursor stoichiometry and interfacial reactions in the PeLEDs and achieve a high external quantum efficiency of 19.6%.

## Introduction

Solution-processed metal halide perovskites have demonstrated great advances in the applications of high-performance light-emitting diodes (LEDs), due to their high photoluminescence (PL) efficiencies, pure and widely tuneable light emission, and excellent charge transport properties^1–5^. In a typical perovskite LED, the luminescent perovskite film is sandwiched between the electron- and hole-injection interlayers with suitable energy levels and electrical properties^6–8^. Both the perovskite active layer and charge-injection layers are of critical importance to achieve high-efficiency perovskite LEDs^3,9,10^. Previous studies have focused on the optimisation of the emissive layer through solution engineering of the perovskite precursors, such as tuning the perovskite dimension, manipulating the precursor composition, incorporating additives or passivation molecules^6,11–14^. However, experience from the development of perovskite solar cells indicates that the surface properties of underlying interlayers also significantly affect the perovskite active layer deposited on top^15,16^. The surface chemical and physical properties of the buried interlayers can affect the perovskite crystallization process, which in turn influences the film quality of the perovskite active layer and consequently the ensuing device performance^17,18^. In addition, the possible reactions between the interlayers and the perovskites even add more complexity^19^. Despite rapid improvements in the external quantum efficiencies (EQEs) of perovskite LEDs during the past few years^1,6,20–22^, an in-depth understanding of the combined effects of the perovskite precursor stoichiometry and the interfacial properties on the resulting perovskite films and devices is yet currently lacking.

Here we investigate perovskite films and LEDs fabricated on commonly used n-type metal oxide electron-transporting layers (ETLs), e.g., zinc oxide (ZnO), titanium oxide (TiO_*x*_) and tin oxide (SnO_2_), modified with an ultra-thin polyethylenimine ethoxylated (PEIE) layer. We demonstrate that incorporating excess organic halides in the precursor greatly suppresses the non-radiative recombination in the resulting films, leading to highly emissive perovskite active layers and efficient LEDs. The deprotonation of excess organic cations by the basic ZnO surface is critical to trigger the transition of precursor intermediates into high-quality perovskite light emitters. We demonstrate that this interface-induced deprotonation process is compatible with further defect passivation strategies. We passivate the perovskite layers with an efficient diamine molecule, 4,7,10-trioxa-1,13-tridecanediamine (TTDDA), and obtain high-efficiency near-infrared (NIR) LEDs with a maximum EQE of 19.6% based on formamidinium (FA)-cesium (Cs) mixed-cation perovskites.

## Results

### Impacts of perovskite precursor stoichiometry

**Fig. 1:**
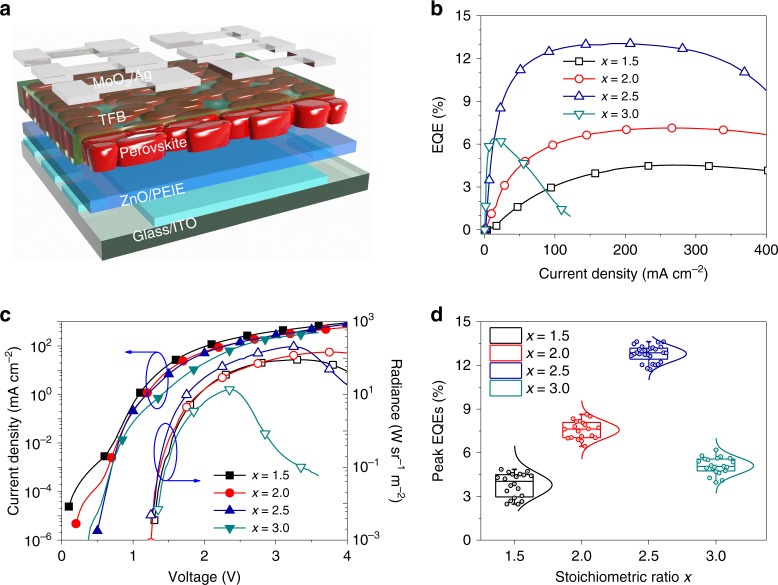
Perovskite LEDs processed from precursors with excess FAI. **a** Schematic diagram of the device architecture of the perovskite LEDs in this work (ITO/ZnO/PEIE/perovskite/TFB/MoO_3_/Ag). **b**–**d** EQE*-J* (**b**), *J & R-V* curves (**c**), and statistical distributions of peak EQEs (**d**) of perovskite LEDs fabricated from precursors with different *x* values (*x* = 1.5, 2.0, 2.5 and 3.0). For all box plots, the line in the center is the median value. The interquartile range (IQR) represents 25th-75th percentile of the EQE values. Whiskers show the extent of the whole EQE values, extending to 1.5 IQR in box-plots

We show the device geometry of our perovskite LEDs in Fig. 1a. ZnO/PEIE and poly[(9,9-dioctylfluorenyl-2,7-diyl)-*co*-(4,4′-(N-(4-sec-butylphenyl) diphenylamine)) (TFB) are used as the electron- and hole-injection layers, respectively^1,6^. We employ a ‘thermally stable’ formamidinium lead triode (FAPbI_3_) perovskite as the light-emitting material, with 10 % of formamidinium iodide (FAI) in the precursor substituted by cesium iodide (CsI) to push the Goldschmidt tolerance factor to a more stable region^23^. We tune the molar ratio (*x*) between (0.9FAI + 0.1CsI) and lead iodide (PbI_2_) (*x* = 1.0, 1.5, 2.0, 2.5 and 3.0) to evaluate the effects of the precursor stoichiometry on the resulting films and devices. In order to focus our investigation on the precursor stoichiometry on its own, no additives or passivation molecules are added in the precursor solutions at this stage. All the perovskite active layers are deposited from a simple one-step spin-coating process and annealed in a glovebox at 100 °C for 10 min (experimental details can be found in the “Methods” section).

We show the impact of increasing amount of organic halides on the device performance in Fig. 1b–d and summarize the device parameters in Supplementary Table 1. We are not able to obtain emitting devices from precursor with the stoichiometric ratio (*x* = 1.0) due to the formation of non-perovskite *δ*-phase in the film (Supplementary Figure 1a), which shows almost no PL emission (Supplementary Figure 1b). The result is consistent with previous reports that a high temperature above 100 °C is generally required to obtain pure *α*-phase FA-Cs mixed-cation iodide perovskites when processed from the stoichiometric precursor without the use of additives or antisolvents^23–25^. We show current density-radiance and voltage (*J* & *R-V*), EQE-current density (EQE*-J*) curves, and histograms of the peak EQEs of the devices prepared from precursors with different *x* values in Fig. 1b–d. We observe enhanced EQEs and radiances with increasing *x* values from 1.5 to 2.5. For the champion device with *x* = 2.5, we obtain a maximum EQE of 13.1 % and a peak radiance of 206.6 W sr^−1^ m^−2^. However, a further increase of *x* to 3.0 results in a declined EQE of 6.4%, along with much lower radiance of 13.6 W sr^−1^ m^−2^ and obvious efficiency roll-off. We notice that the current density from the *x* = 3.0 device is much lower than those processed from precursors with smaller *x* values (Fig. 1c). The existence of a large amount of excess organic halides in this case is likely to affect the electronic properties of the resulting perovskites and introduce barriers for charge injection into the films, resulting in the decreased EQE and obvious efficiency roll-off^26^.

**Fig. 2:**
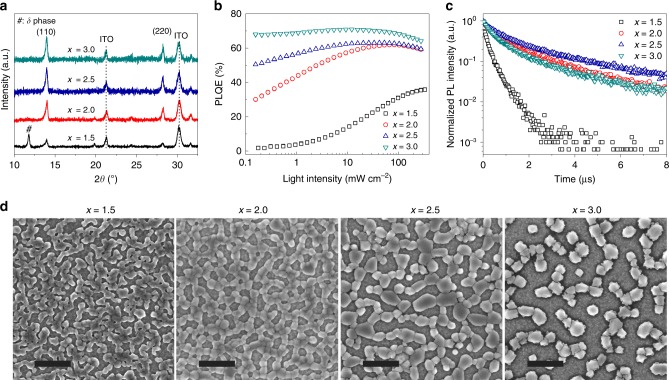
Perovskite films processed from precursors with different component ratios. **a**–**d** XRD patterns (**a**), light-intensity-dependent PLQEs (**b**), PL decays (**c**) and SEM images (**d**) of thermal annealed perovskite films processed from precursors with different *x* values (*x* = 1.5, 2.0, 2.5 and 3.0). The scale bars in the SEM images are 1 µm

We perform a range of the film characterizations to understand the effects of excess organic components on the film quality and the ensuing device performance. As the X-ray diffraction (XRD) patterns of the perovskite films show in Fig. 2a, we observe a clear evolution of the perovskite crystal structures from a mixture of *α/δ*-phase to pure *α*-phase with increasing *x* values from 1.5 to 3.0. The result is consistent with a previous study showing that excess FAI in the precursor is helpful for the formation of *α*-phase perovskite at a low processing temperature^27^.

The films with higher *x* values show much enhanced PL intensity, suggesting that excess organic halides effectively passivate the defects in the resulting films (Supplementary Figure 2a). According to the light-intensity-dependent PL quantum efficiency (PLQE) results in Fig. 2b, we observe suppressed non-radiative recombination in perovskite films processed with increasing organic halides. High PLQEs of over 50 % are obtained for films (*x* = 3.0 and 2.5) under excitation of different light intensities from 0.15 to 300 mW cm^−2^. In contrast, the PLQEs of perovskite films processed from precursors with smaller *x* values (*x* = 1.5 and 2.0) are much lower under low excitation intensities and keep increasing with increasing excitation intensities, indicating a trap filling process due to the existence of defects in the perovskite films^28^. We further conduct time-correlated single photon counting (TCSPC) measurements to investigate the charge carrier decay dynamics (Fig. 2c). Consistent with the PLQE results, the PL lifetime increases with the *x* value increasing from 1.5 to 2.5. Considering the further improved PLQE value in the *x* *=* 3.0 sample, the slight decrease of the PL lifetime in this case is ascribed to accelerated radiative recombination^13^, rather than the existence of more defects.

Different precursor stoichiometry also results in morphology changes in the obtained perovskite films. From the scanning electron microscopy (SEM) measurements (Fig. 2d), we observe reduced film coverage for perovskite films prepared from precursors with increasing *x* values. This non-continuous perovskite film morphology was demonstrated beneficial to improve the light-outcoupling efficiency^1^. However, the perovskite film processed from the *x* = 3.0 precursor shows dramatically low film coverage, which is possibly one of the reasons for the strong efficiency roll-off under high current densities in the resulting perovskite LEDs.

### Effects of the buried interface on the perovskite films

Having demonstrated the importance of the precursor stoichiometry for the fabrication of high-efficiency perovskite LEDs, however, we cannot obtain working devices when we replace the ZnO with two other n-type metal oxide thin films that are commonly used in perovskite optoelectronic devices, i.e., TiO_*x*_ and SnO_2_^29,30^.

**Fig. 3:**
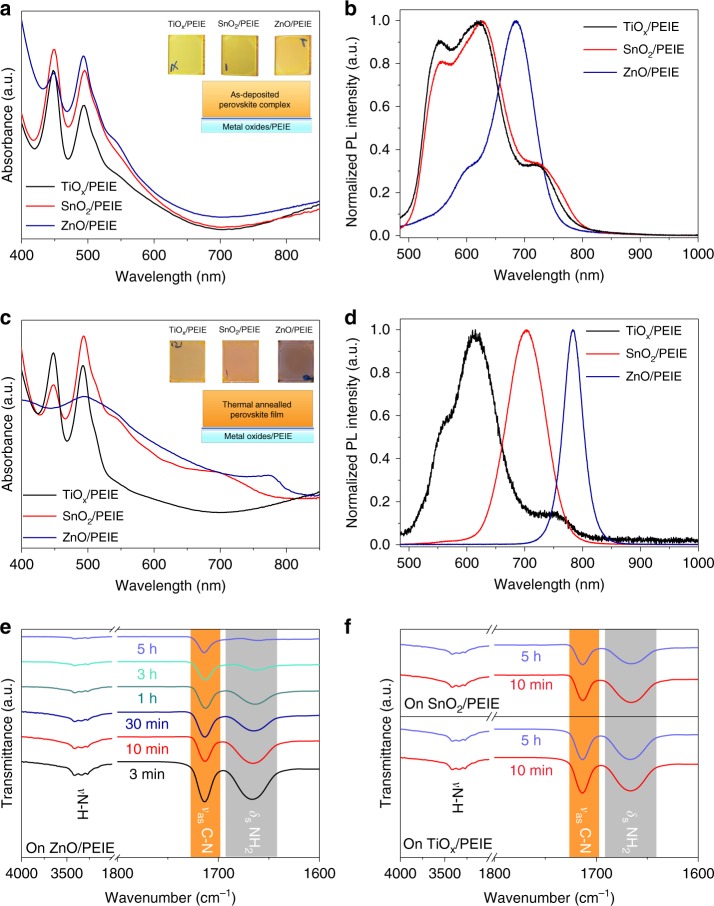
Optical characteristics of perovskite films (*x* = 2.5) on different substrates. **a**–**d** UV-Vis absorption (**a**, **c**) and normalized PL spectra (**b**, **d**) of the perovskite films on TiO_*x*_/PEIE, SnO_2_/PEIE and ZnO/PEIE substrates, respectively before (**a**, **b**) and after the thermal annealing (**c**, **d**). Insets show the corresponding photographs of the perovskite films on different substrates. **e**–**f** FTIR spectra of perovskite films on ZnO /PEIE (**e**), TiO_*x*_/PEIE and SnO_2_/PEIE (**f**) substrates with different annealing time

We now proceed to understand the reasons why different metal oxides result in significant difference in the device performance. Considering similar electronic properties of the three metal oxides and the same hole injection contact on top, we focus on the perovskite layers on different metal oxides. We first characterize the as-deposited precursor films (*x* = 2.5) on different substrates (before the thermal annealing), and observe two strong exciton absorption peaks at around 450 nm and 500 nm from the ultraviolet-visible (UV-Vis) absorption spectra (Fig. 3a). The result is consistent with previously demonstrated ground-state excitations from the low-dimensional coordinated intermediate complexes containing excess methylammonium iodide (MAI), e.g., one-dimensional (1D) perovskite chains or self-assembled two-dimensional (2D) perovskite sheets in the as-deposited precursor films^31,32^.

We believe that the formation of these intermediate complexes is caused by the introduced excess organic halides, which hinder the sharing of iodides between different lead polyiodides octahedral, resulting in colloids with decreased sizes in the perovskite precursors. The different colloid sizes in the precursors with different *x* values are confirmed by the dynamic light scattering (DLS) results (Supplementary Figure 3), which show a decreasing trend from around 163.0 nm (*x* = 1.0) to around 3.6 nm (*x* = 3.0). We also note that different substrates already result in difference in these intermediate complexes. For the perovskite on the ZnO/PEIE substrate, we observe an additional absorption shoulder locating at around 550 nm, which may origin from the soft-packing or self-assembly of the intermediate phases in the complex films^31^. The emergence of this absorption shoulder indicates aggregation of the intermediate phases, consistent with the obvious red-shift in the PL emission (Fig. 3b). These results indicate that the perovskite precursor complexes on the ZnO/PEIE show more obvious aggregation right after the spin-coating or during the film drying process, which is different from those on the other two substrates.

After thermal annealing (*x* = 2.5), we observe negligible difference in the main diffraction peaks of the XRD results of the three samples, all showing pure *α*-phase perovskite features (Supplementary Figure 4)^27^. The perovskite film on ZnO/PEIE shows enhanced diffraction intensity, suggesting improved crystallinity of the film. Despite similar XRD results, we observe significant difference in the absorption and emission spectra of the perovskite films on different substrates (Fig. 3c, d). We observe typical absorption and PL spectra of the *α*-phase perovskites from the films on ZnO/PEIE. In contrast, both the absorption and PL spectra of the films on TiO_x_/PEIE show negligible changes after thermal annealing, as compared with those of the intermediate phases in the as-deposited precursor complexes. For perovskites on SnO_2_/PEIE, although we observe redshift of the absorption and PL spectra as compared with the precursor complexes, both the absorption and emission peaks locate at higher energies compared with the film on the ZnO/PEIE substrate. Difference in films on the three substrates is also clearly visible from the optical images of the resulting perovskite films (insets of Fig. 3a, c). The result suggests that the films deposited on SnO_2_/PEIE and TiO_x_/PEIE mostly retain their intermediate phases, which are in high disorder. These intermediate phases show no obvious diffraction peaks in the XRD patterns, but are discernible from the optical characterizations. We conclude that, under the relatively low processing temperature of 100 °C we use here, the efficient transition from precursor complexes to pure-phase and highly luminescent perovskite crystals only happens on the ZnO/PEIE substrate.

### Role of FA^+^ deprotonation for perovskite crystallization

The different electrical, structural and optical properties of the resulting perovskite films suggest that the perovskite crystallization is highly related to the buried substrates. We notice that the three n-type metal oxides exhibit different surface chemical properties. The measured isoelectric point (IEP) values, which represent the surface acidity of the metal oxide films, are reported to be 3.5–6.2 for TiO_*x*_ (acidic), 6.6–9.5 for SnO_2_ (neutral) and 8.7–10.3 for ZnO (basic), respectively^33^. As demonstrated by previous theoretical calculations and experimental results, the organic cations may experience a deprotonation process upon in contact with metal oxide films. The deprotonation probability depends on the surface chemical properties of the metal oxide, of which a basic surface with a higher IEP value is likely to induce an easier deprotonation of the organic cations^19,34^. We anticipate that the ultra-thin PEIE layer used here cannot completely prevent the direct contact and interactions between the perovskite complexes and metal oxides. This is also consistent with the observed absorption shoulder at around 550 nm and the red-shifted PL spectrum of the as-deposited precursor complex on ZnO/PEIE, resulting from the interaction between perovskites and ZnO (Fig. 3a, b).

To further explore the difference of substrates-induced deprotonation of the organic cations, we conduct the Fourier transform infrared (FTIR) spectroscopy measurements to monitor the evolution of the FAI molecules during the perovskite crystallization. We first collect the FTIR spectra of the pristine FAI and the perovskite films processed from precursor solutions with different *x* values on ZnO/PEIE. In the pristine FAI film, the peaks locating at 3420–3260, 1714 and 1666 cm^−1^ can be ascribed to N–H stretching (*ν* N–H), C–N stretching (*ν*_as_ C–N) and –NH_2_ scissoring vibrations (*ν*_s_ NH_2_), respectively (Supplementary Figure 5a)^35^. In the perovskite film processed from the stoichiometric precursor (*x* = 1.0), the *ν*_s_ NH_2_ is greatly restricted because of the formation of N-H^…^I hydrogen bonds within the crystal structure^36,37^. The *ν*_s_ NH_2_ becomes more obvious under organic cation-rich conditions (*x* = 2.0 and *x* = 3.0) due to the existence of FAI molecules in the adjacent area outside of the perovskite crystals (Supplementary Figure 5b)^1,38^. Therefore, the relative peak intensity of *ν*_s_ NH_2_ at 1666 cm^−1^ can be used to monitor the amount of excess FAI molecules within the films. As shown in Fig. 3e, we observe a continuous decrease in the intensity of *ν* N–H and *ν*_as_ C–N of FAI molecules on ZnO/PEIE, indicating a continuous deprotonation of the excess FA^+^ cations in the perovskite films during the thermal annealing^35^. Meanwhile, the *ν*_s_ NH_2_ at 1666 cm^−1^ shows a faster decline as compared with that of *ν*_as_ C–N, suggesting that the FA^+^ cations outside the crystal lattice experience a faster deprotonation rate during the film crystallization (Supplementary Figure 6a). On the contrary, the FTIR results of the perovskite films show almost no change on TiO_x_/PEIE or a slight decrease on SnO_2_/PEIE substrate after 5 h thermal annealing (Fig. 3f and Supplementary Figure 6b). We observe slight interfacial reactions of the organic cations on SnO_2_/PEIE due to a higher IEP value of SnO_2_ compared to that of TiO_*x*_, suggesting that the recently observed thermal instability of perovskite solar cells based on bare SnO_2_ is also possibly related to the potential reaction at the perovskite/SnO_2_ interface^39^. These FTIR results are consistent with the reported IEP values of the metal oxides and further demonstrate the easier deprotonation of FA^+^ cations on basic metal oxide substrates, e.g., ZnO in our case.

**Fig. 4:**
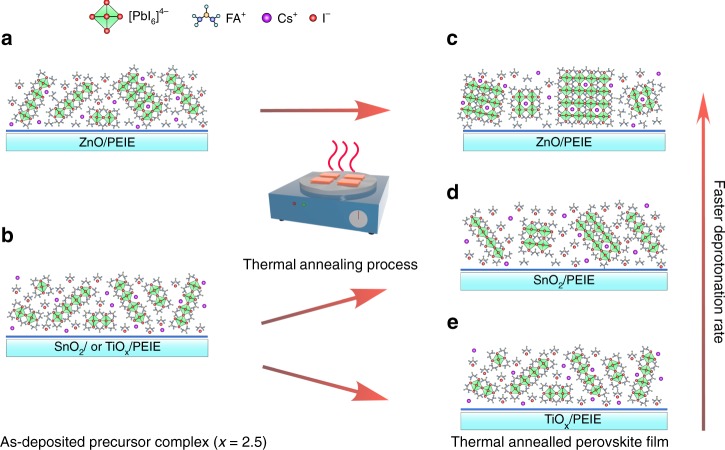
Schematic illustration of perovskite (*x* = 2.5) crystallization on different substrates. **a**, **b** The as deposited perovskite precursor complexes on ZnO/PEIE (**a**) and on TiO_x_/PEIE or SnO_2_/PEIE (**b**) substrates. **c**–**e** The thermal annealed perovskite films on ZnO/PEIE (**c**), SnO_2_/PEIE (**d**) and TiO_*x*_/PEIE (**e**) substrates, respectively

We now obtain a clear picture of the crystallization process and film properties of the perovskite films on the three substrates. As we show in Fig. 4, in the as-deposited precursor complexes (organic components-rich condition, e.g., *x* = 2.5), most of the excess FAI exist as molecules in the form of ionic pairs with strong hydrogen bonds between N–H^…^N. The corner sharing [PbI_6_]^4−^ units are surrounded by the abundant organic halides, which inhibit the formation of 3-Dimentional (3D) perovskite phases, easily generating low-dimensional intermediate phases on the substrates (Fig. 4a, b). Considering the high decomposition temperature of FAI (246 °C), the mild thermal annealing condition (100 °C) we used here is not able to decompose and eliminate the abundant FAI molecules on the SnO_2_/PEIE and TiO_x_/PEIE substrates. This consequently hinders the transition of intermediate phases to high-quality perovskite crystals during the thermal annealing process (Fig. 4d, e)^36^. In contrast, the basic ZnO/PEIE interface induces efficient deprotonation of the organic cations, providing external energy to remove the FAI molecules during the thermal annealing. We note that, even after prolonged thermal annealing process (100 °C for up to 5 h), the films on SnO_2_/PEIE and TiO_x_/PEIE substrates remain at their intermediate states as evidenced by the photographs, UV-vis absorption and PL spectra (Supplementary Figure 7a–c), apart from slightly enhanced crystallinity according to the XRD patterns (Supplementary Figure 7d). We anticipate that the ZnO-induced efficient deprotonation of the organic cations removes the crystallization barriers, reduces the activation energy for perovskite crystallization, and hence promotes the phase transition from the intermediate phases to *α*-phase perovskite crystals, generating high-quality emissive perovskite active layers (Fig. 4c).

We note that, the thermal annealing of perovskite films on ZnO/PEIE substrates needs to be carefully controlled to obtain optimized crystalline emitters without further decomposing the as-formed perovskite active layer. From the XRD patterns of perovskite films with different annealing time (Supplementary Figure 8), we observe no PbI_2_ (11.8°) from the films with 30 min annealing. However, further extending the annealing time to 1 h results in the formation of obvious PbI_2_ in the film^19^. These results indicate that at the beginning of the thermal annealing, the deprotonation process by ZnO/PEIE substrates mainly contributes to phase transition rather than the perovskite decomposition. However, a long-term annealing of the perovskite film on ZnO/PEIE causes decomposition of the as-formed perovskite active layer due to the continuous deprotonation of the FAI by the basic ZnO surface.

### Perovskite films and LEDs with further defect passivation

Having revealed the critical role of the perovskite precursor stoichiometry and the unique interfacial reactions at the ZnO/perovskite interfaces, it is natural for us to investigate whether our finding is compatible with state-of-the-art molecular passivation that has boosted the device performance. A diamine passivation molecule, i.e., 4,7,10-trioxa-1,13-tridecanediamine (TTDDA), which was recently proven efficient in reducing the defect states in the perovskite films, is incorporated in the precursor solutions^38^. In the FTIR spectra of the resulting perovskite films, we observe a distinct C-H stretching (*ν* C-H) at 2865 cm^−1^ from the TTDDA (Supplementary Figure 9), which exists in neither the neat FAI film nor the pristine perovskite film, confirming the existence of TTDDA molecules in the resulting perovskite films. As a result of the TTDDA passivation, the non-radiative recombination in perovskite film is further suppressed, as indicated by improved PLQE values up to around 70 % and prolonged charge carrier lifetime (Supplementary Figure 10a, b).

We notice that the incorporation of TTDDA results in neither low-dimensional phases nor impurities (Supplementary Figure 10c, d). With the same film processing conditions, we obtain highly luminescent perovskite films on the ZnO/PEIE substrate while the formation of *α*-phase perovskite on SnO_2_/PEIE and TiO_x_/PEIE substrates is still hindered as evidenced by the absorption and PL results (Supplementary Figure 11). These results further demonstrate the critical role of the basic ZnO/PEIE substrate on promoting the perovskite crystallization during the film formation process.

**Fig. 5:**
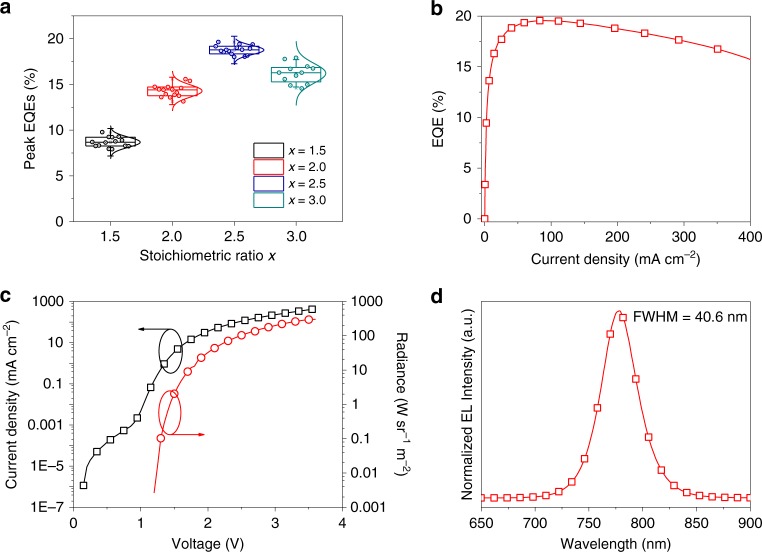
Device performance of perovskite LEDs with TTDDA passivation. **a** Statistical distributions of peak EQEs of perovskite LEDs fabricated from TTDDA-containing precursors with different component ratios. **b**, **c** EQE*-J* (**b**), *J & R-V* (**c**) curves of the champion device with TTDDA passivation. **d** Normalized EL spectrum of the champion device operated at an applied voltage of 3.5 V. For all box plots, the line in the center is the median value. The interquartile range (IQR) represents 25th-75th percentile of the EQE values. Whiskers show the extent of the whole EQE values, extending to 1.5 IQR in box-plots

We fabricate perovskite LEDs with the TTDDA passivation and show the device results in Fig. 5a and Supplementary Figure 12. Consistent with the devices without TTDDA, we observe the same trend in the device efficiency, with the *x* = 2.5 devices giving the highest performance. The champion device with TTDDA passivation shows a peak EQE of 19.6 % along with a high radiance of 301.8 W sr^−1^ m^−2^ (Fig. 5b, c). In addition, the typical electroluminescence (EL) spectrum of the champion device shows a narrow emission at around 780 nm with a fullwidth at half-maximum (FWHM) of around 40 nm, which represents one of the narrowest EL emission spectra in NIR region (Fig. 5d)^6,21^. We further characterize the device operational stability, which is very important for practical applications of LEDs (Supplementary Figure 13). We find that the device based on the TTDDA-passivated perovskite film achieves a *T*_50_ lifetime (the time for device to decay to 50 % of its peak EQE value) of 7 h under a constant current density of 20 mA cm^−2^.

## Discussion

The gathered information on the synergistic effects of the precursor stoichiometry and the deprotonation of the organic cations by the basic ZnO agrees well with previous efficient LEDs based on methylammonium (MA), FA or hybrid multiple-quantum-wells (MQWs) structure perovskites, especially those with ZnO/PEIE as the bottom electron transport layer^1,6,26,34^. In order to demonstrate the generality of the deprotonation on promoting the perovskite film growth, we prepare MA-based perovskite films (MAI:PbI_2_ = 2.5) on the three different substrates. We observe typical UV-vis absorption and high crystalline feature of pure-phase MAPbI_3_ on the ZnO/PEIE substrate (Supplementary Figure 14). Similar to the FAPbI_3_, the MAPbI_3_ perovskite films deposited on TiO_*x*_/PEIE and SnO_2_/PEIE exhibit optical properties of the intermediate states, with no crystalline perovskites formed after the thermal annealing.

We believe that the results in this study are general for the fabrication of the 2D, 3D or quasi-2D perovskite films that contain organic cations, which can be deprotonated by the basic ZnO surface during the processing conditions. In previous studies, the enhanced device performance of perovskite LEDs fabricated on ZnO/PEIE was ascribed to improved surface wettability or reduced work function of the substrates^40^. However, in light of our findings in the present study, we believe that the precursor stoichiometry and the unique interfacial reactions during the perovskite crystallization induced by basic ZnO play very important roles in achieving high device performance.

It has been demonstrated that a continuous deprotonation reaction of the perovskite film by ZnO is harmful to the film and device stability, especially under an elevated temperature during the device operation. To further demonstrate the effect of interfacial interactions on the stability, we carry out in-situ PL measurements of the perovskite film on ZnO/PEIE. A recent report revealed that the temperature of a working perovskite LED based on mixed-cation perovskite of (Cs_0.2_FA_0.8_)PbI_2.8_Br_0.2_ could reach 41 °C under a constant current density of 40 mA cm^−2^, ref. ^41^. We measure the evolution of the PL emission from the perovskite film at 60 °C, which represents a reasonably high temperature during device operation. We observe no change of the PL emission peak and slight increase of the PL intensity after 17 h heating (Supplementary Figure 15), suggesting that the interfacial reactions between the ZnO/PEIE and the perovskite active layer are negligible under this mild temperature. The degradation mechanisms and critical factors affecting the device stability require further investigations.

In summary, we have demonstrated the critical role of the synergistic effect of the precursor stoichiometry and the interfacial reaction on achieving high-efficiency perovskite LEDs. We have observed efficient passivation of the excess organic halide on the defects in perovskite films. More importantly, deprotonation of these excess organic cations by a buried surface with a higher IEP value is beneficial to promote the transition of the intermediate phases into high-luminescence perovskite layers. In addition, the deprotonation process is compatible with state-of-the-art passivation molecules. We believe that the key findings in this work provide insights for further development of high-performance perovskite LEDs, and offer inspirations for controllable deposition of the perovskite films for other optoelectronic devices on substrates with different surface properties.

## Methods

### Perovskite precursor solutions

The perovskite precursor solutions were prepared by mixing FAI, CsI and PbI_2_ with a molar ratio of *x* in N, N-Dimethylformamide (DMF, anhydrous) at a concentration of 0.115 M (PbI_2_) ((0.1CsI + 0.9FAI): PbI_2_ = *x*, *x* = 1.0–3.0). For the precursor with TTDDA passivation molecules, 17.2 mM TTDDA was added into the solution. MA-based perovskite precursor solution with a concentration of 0.15 M was prepared by dissolving MAI and PbI_2_ with a molar ratio of 2.5:1 in DMF. All the prepared precursor solutions were stirred at 60 °C for over 2 h before the film preparation.

### Substrates preparation

ITO coated glass substrates were washed in the detergent aqueous solution prior to 10 min UV-ozone treatment. Tin (IV) oxide (SnO_2_) colloidal solution and titanium oxide (TiO_*x*_) precursor solution were prepared according to previous literatures^42,43^. Typically, the SnO_2_ colloidal solution was purchased from Alfa Aesar (15 wt% in water colloidal dispersion) and was diluted to 3 wt% with deionized water for film deposition. TiO_x_ precursor solution was prepared by diluting the titanium iso-propoxide (2 M, 750 µL) in iso-propanol (IPA, 10 mL) under vigorous stirring condition. After that, 70 µL HCl solution (2 M) was added drop-wisely to the solution. The solution was further stirred for 12 h before use. ZnO nanoparticles were synthesised by a solution precipitation method^44^. Typically, 1.5 mmol zinc acetate hydrate (Zn(Ac)_2_·2H_2_O) in 15 mL dimethyl sulfoxide (DMSO) was stirred at 30 °C. Then 2.8 mmol tetramethylammonium hydroxide pentahydrate (TMAH·5H_2_O) in 5 mL ethanol was dropped in Zn(Ac)_2_·2H_2_O solution. The solution was kept stirring for 24 h prior to the precipitation process. The ZnO nanoparticles were precipitated by ethyl acetate, and washed with ethanol and ethyl acetate for one more time. Finally, the obtained ZnO nanoparticles were dispersed in ethanol (8 mL) and filtered with a 0.45 µm PTFE filter before use. The PEIE dispersion was diluted in IPA solution with a concentration of 0.05 wt%. The metal oxide films were prepared by spin-coating the solution on ITO substrates in ambient conditions. For SnO_2_ and TiO_x_ substrates, the films were annealed at 150 °C for 30 and 90 min, respectively. PEIE was spin-coated on top of the metal oxide films at 5000 r.p.m. for 30 s and then annealed at 100 °C for 10 mins in the glove-box.

### Device fabrication and characterization

Perovskite LEDs in this work were fabricated in a nitrogen filled glove-box. The perovskite films were spin-coated at 3000 r.p.m. for 30 s and then annealed on a pre-heated hotplate at 100 °C for 10 min. TFB (12 mg ml^−1^ in chlorobenzene) layer was spin-coated on top of the perovskite film at 3000 r.p.m. for 30 s. After that, 7 nm MoO_3_ and 100 nm Ag were deposited as the electrode in the thermal evaporator. The device active area is 7.25 mm^2^. The fabricated perovskite LEDs were measured in a glove-box at room temperature. A Keithley 2400 source meter was used to collect the current density and the driving voltage. An integrating sphere together with a QE Pro spectrometer (Ocean Optics) were used to collect the light emission. The applied voltage started from 0 V and increased with a step of 0.05 V, lasting for 300 ms at each voltage step for stabilisation and measurements. The integrating sphere-spectrometer system was calibrated by a Vis-NIR radiometric calibration sources for absolute light intensity and a HG-1 calibration source for wavelength (Ocean Optics). The operational lifetime measurements of the LEDs were conducted using the same setup under a constant current density of 20 mA cm^−2^. The layout of the setup for LED measurements is displayed in Supplementary Figure 16.

### Precursor and perovskite film characterizations

Size distributions of the colloids in perovskite precursor were measured by a zeta potential analyser (Brookhaven ZetaPlus). Morphology images of the perovskite films were obtained by a FEI (Quanta 200 FEG) scanning electron microscopy under a voltage of 5 kV. X-ray diffraction (XRD) patterns were collected using an X-ray diffractometer (Panalytical X’Pert Pro) with CuK*α* radiation. The X-ray was generated on a Copper target and the wavelength of the X-ray was 1.54 Å.

Ultraviolet-visible (UV-Vis) absorbance spectra were obtained with a PerkinElmer Lambda 900 in transmission mode. Steady state PL spectra were obtained with a 450 nm excitation laser and an Andor spectrometer (Shamrock sr-303i-B, coupled to a Newton EMCCD Si array detector). The intensity-dependent PLQE results of perovskite films were obtained by an integrated system of a 450 nm continuous wavelength laser, optical fiber, spectrometer and integrating sphere. Time-correlated Single Photon Counting **(**TCSPC) PL decays were obtained by using an Edinburgh Instruments spectrometer (FLS980) with a 633-nm pulsed laser (less than 100 ps, 0.1 MHz, YSL Supercontinuum Source SC-PRO). The excitation laser intensity was set at 0.5 mW cm^−2^). In-situ PL spectra of perovskite films were obtained with a QE Pro spectrometer (Ocean Optics). The spectra were collected right after placing the samples on a pre-heated hotplate. A 450 nm LED laser was used to excite the film samples.

Fourier-transform infrared (FTIR) spectra of perovskite films were recorded by a PIKE MIRacle Vertex 70 Spectrometer (Bruker) using a DLaTGS detector at room temperature. The spectrometer is equipped with a PIKE MIRacle^TM^ attenuated total reflectance (ATR) accessory as the sampling accessory. The measuring system was continuously purged with N_2_ before and during the measurements. The spectra were acquired at 2 cm^−1^ resolution with 30 scans over a wavenumber range between 4000 and 1000 cm^−1^. As for the perovskite films on different substrates, background spectra were obtained by measuring SnO_2_/PEIE_,_ TiO_x_/PEIE and ZnO/PEIE substrates. The presented spectra were baselined-corrected by subtracting a linear baseline over the spectral ranges.

## Supplementary information

Supplementary Information

## Data Availability

The datasets generated and/or analysed during the current study are available from the corresponding author on reasonable request.
