# Supplementary material for: Unveiling the synergistic effect of precursor stoichiometry and interfacial reactions for perovskite light-emitting diodes
Source: Nat Commun. 2019 Jun 27;10:2818. doi: 10.1038/s41467-019-10612-3 (PMC6597563; doi:10.1038/s41467-019-10612-3)
Supplement: Supplementary file 1 — Supplementary Information [file 41467_2019_10612_MOESM1_ESM.pdf]

Supplementary Information

Unveiling the synergistic effect of precursor stoichiometry and interfacial reactions for perovskite light-emitting diodes

Yuan et al.

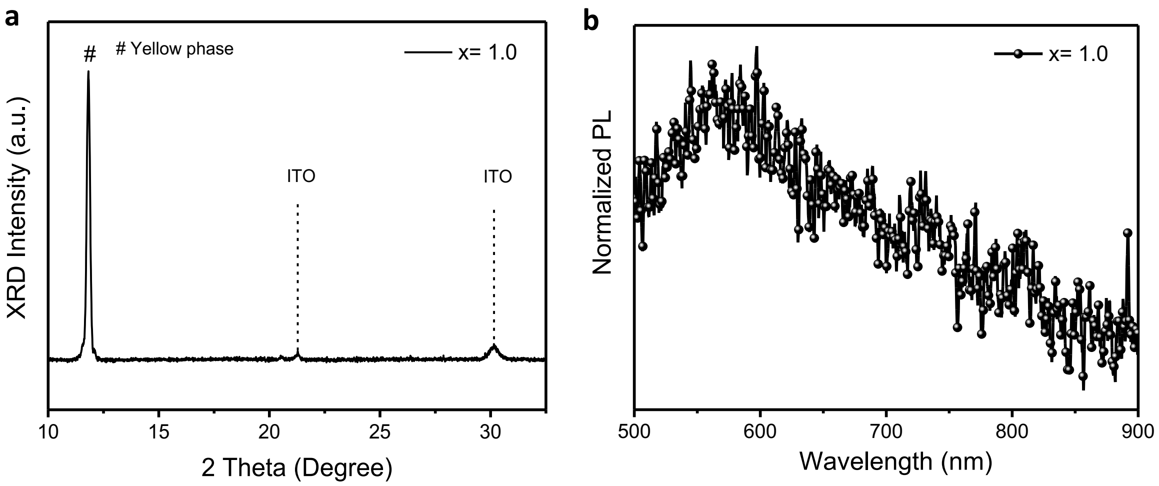

Supplementary Figure 1. Perovskite film processed from stoichiometric precursor. a-b, XRD pattern (**a**) and PL spectrum (**b**) of perovskite film processed from precursor solution with stoichiometric ratio (x=1).

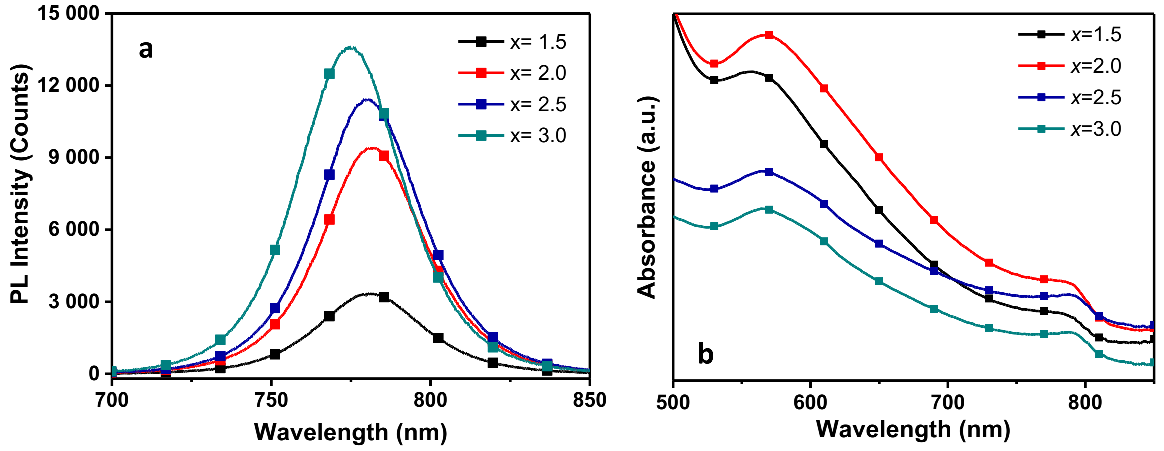

Supplementary Figure 2. Optical properties of perovskite films. a-b, PL (**a**) and UV-vis absorption spectra (**b**) of perovskite films processed from precursors with different *x* values (x = 1.5, 2.0, 2.5 and 3.0) after thermal annealing.

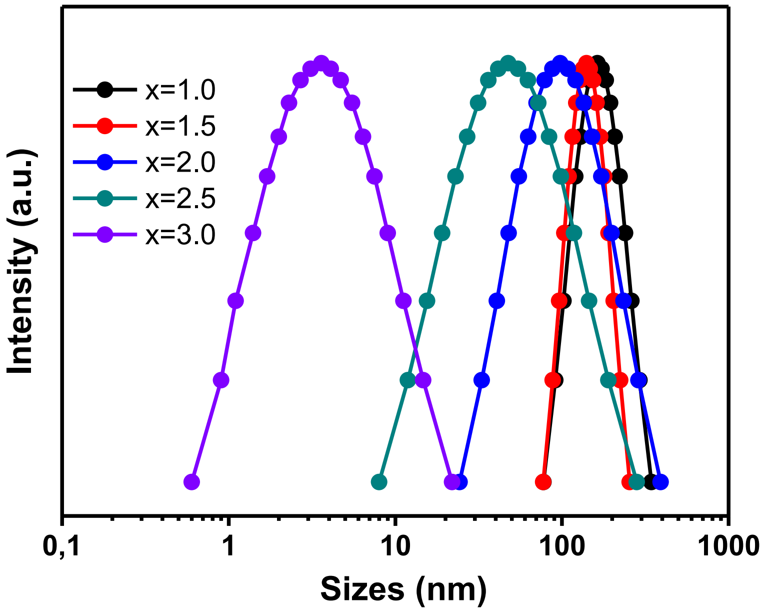

Supplementary Figure 3. Sizes of colloids in the perovskite precursors. Dynamic light scattering (DLS) results of the mixed-cation perovskite precursors with different *x* values.

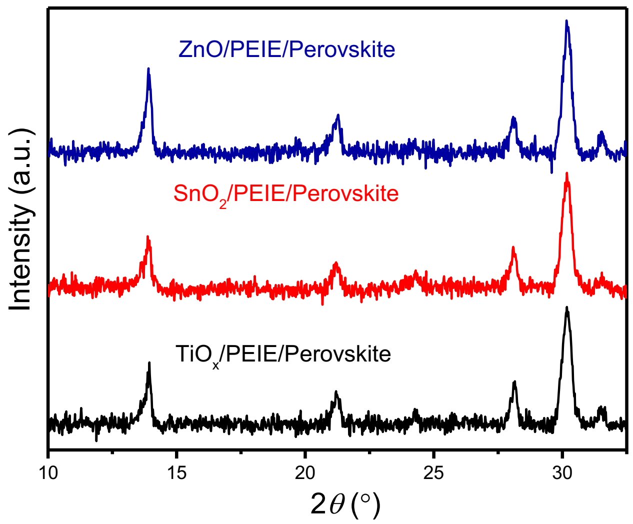

Supplementary Figure 4. Crystallinity of perovskite films on different substrates. XRD patterns of thermal annealed (100 ºC for 10 min) perovskite films (x = 2.5) on TiO_x/PEIE, SnO_2/PEIE and ZnO/PEIE substrates.

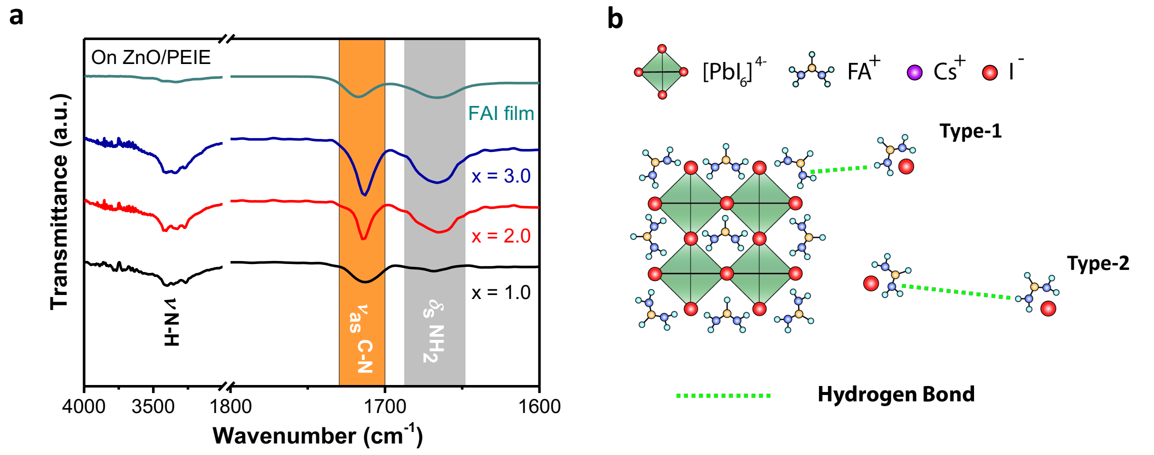

Supplementary Figure 5. FTIR characterization results. a, FTIR spectra of pristine FAI and perovskite films with different *x* values on ZnO/PEIE substrates. **b**, Formation of the hydrogen bond in the perovskite film: Type-1: the FAI molecules stay at the surface of the crystals form hydrogen bonds with free-standing FAI in the neighbouring area. Type-2: the free-standing FAI molecules form hydrogen bonds with each other.

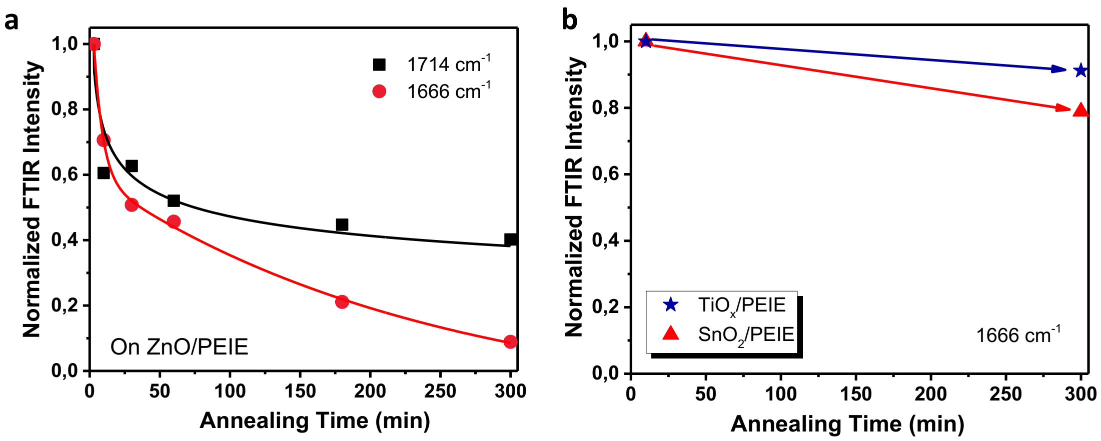

Supplementary Figure 6. Evolution of FTIR peak intensity of perovskite film on different substrates. a, Intensity evolution of v_s NH_2 (1666 cm^{-1}) and v_{as} C-N (1714 cm^{-1}) with the annealing time for perovskite films on the ZnO/PEIE substrate. **b**, Intensity of v_s NH_2 (1666 cm^{-1}) after different annealing time for perovskite films on TiO_x/PEIE and SnO_2/PEIE substrates.

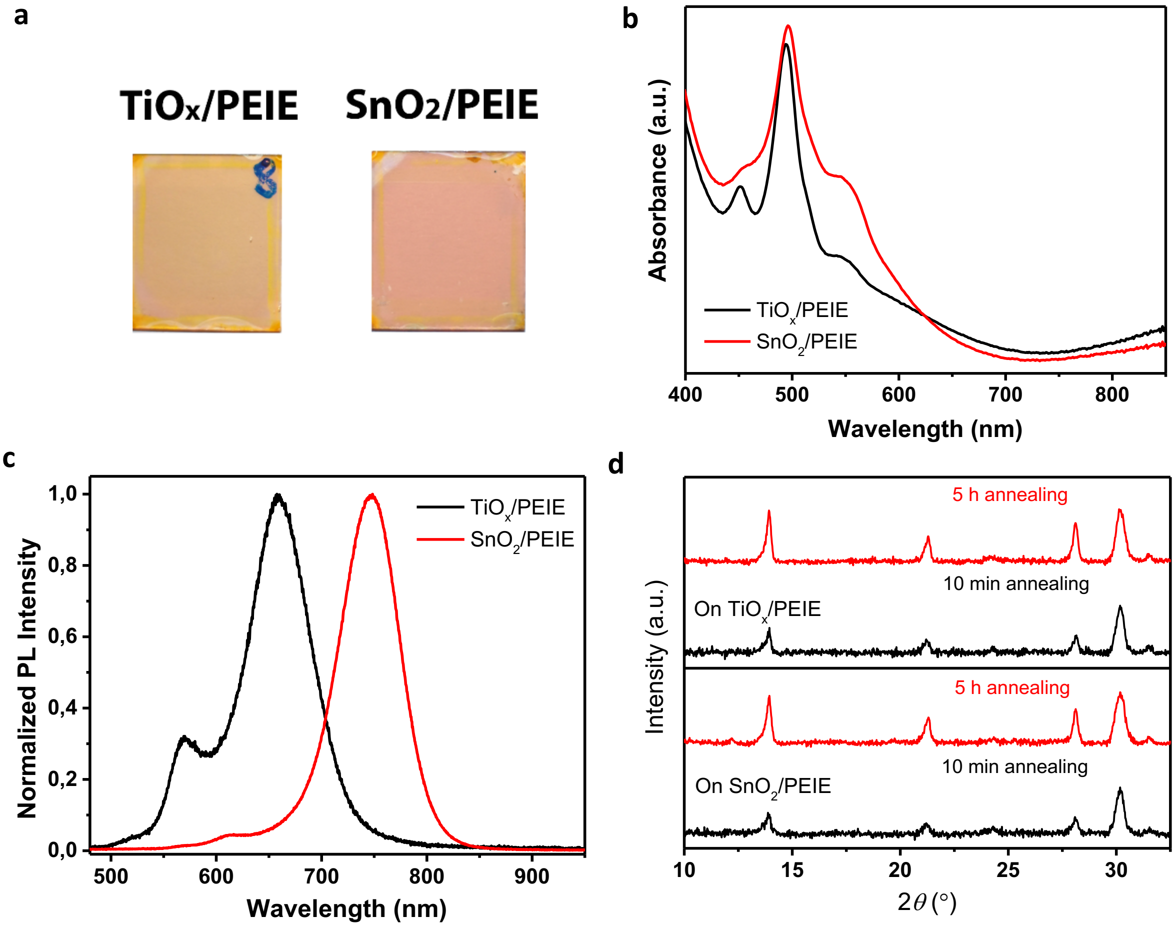

Supplementary Figure 7. Films on TiO_x/PEIE and SnO_2/PEIE with prolonged annealing. **a-c,** Photographs (**a**), UV-vis absorption spectra (**b**) and normalized PL spectra (**c**) of perovskite films (x = 2.5) that annealed for 5 h on TiO_x/PEIE and SnO_2/PEIE substrates. **d**, XRD patterns of the perovskite films on TiO_x/PEIE and SnO_2/PEIE substrates after 10 min and 5 h thermal annealing.

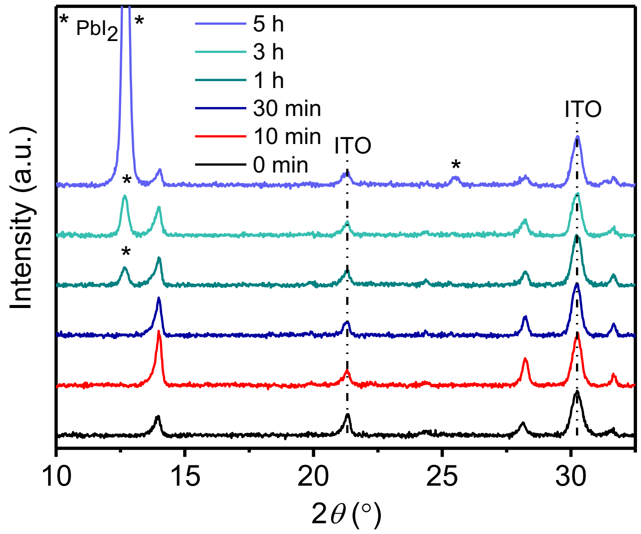

Supplementary Figure 8. Perovskite film on ZnO/PEIE substrate. XRD patterns of the as-deposited precursor complex (x = 2.5, 0 min) and the obtained perovskite films with different annealing time.

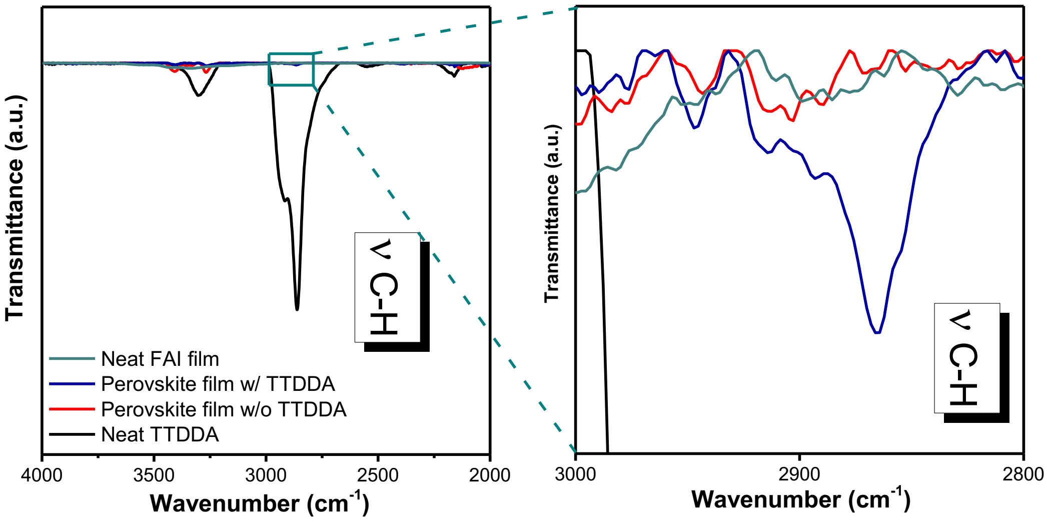

Supplementary Figure 9. FTIR spectra of neat TTDDA, FAI films, perovskite films (*x* = 2.5) with and without TTDDA passivation. The FTIR peak locating at 2870 cm^{-1} is ascribed to the stretching of C-H (ν C-H) from the DDTTA chain. The right one is the zoom-in result of the FTIR spectra in the range of 2800-3000 cm^{-1}.

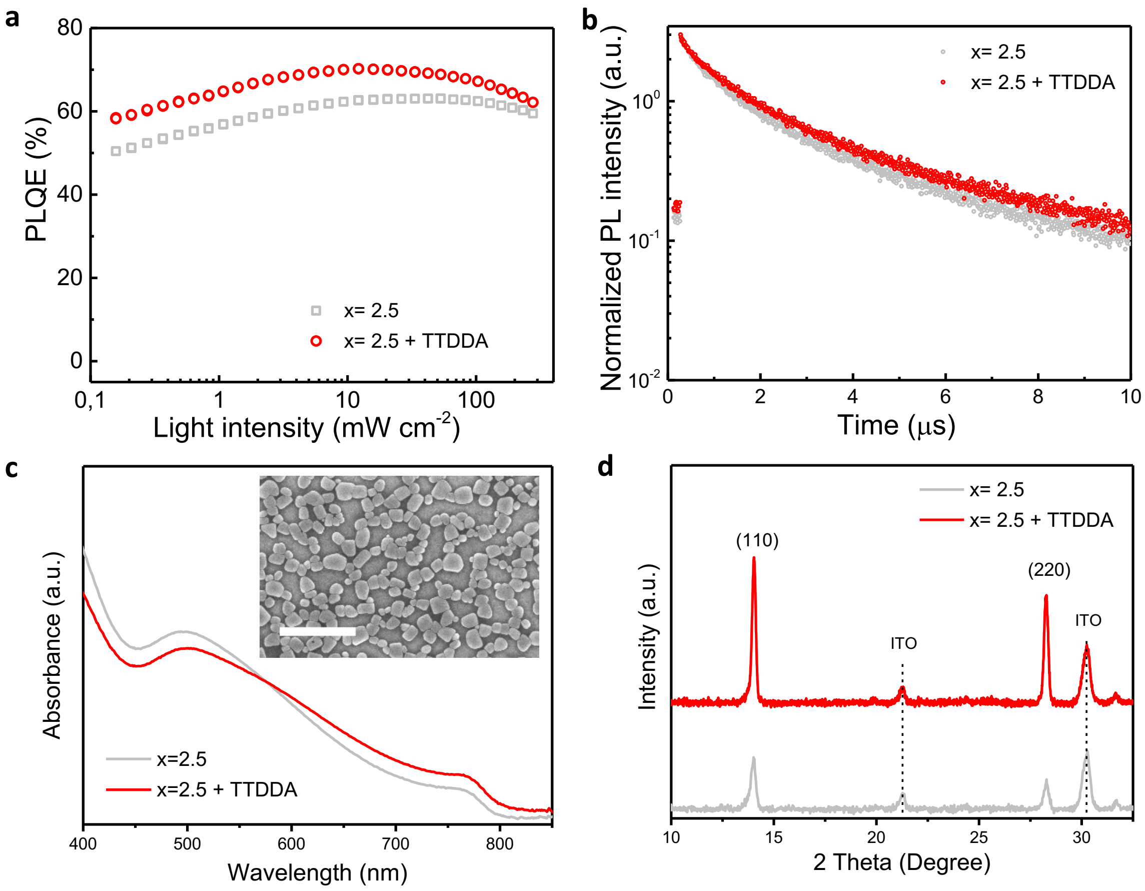

Supplementary Figure 10. Characterizations of perovskite film with TTDDA passivation. **a-d**, Light-intensity dependent PLQEs (**a**), PL decay curves (**b**), UV-vis absorption spectra (inset shows the SEM image) (**c**) and XRD patterns **(d)** of perovskite films (x = 2.5) with TTDDA passivation. The results of pristine perovskite films without TTDDA are shown as the grey curves for comparison. Scale bar in the inset is 2 μm.

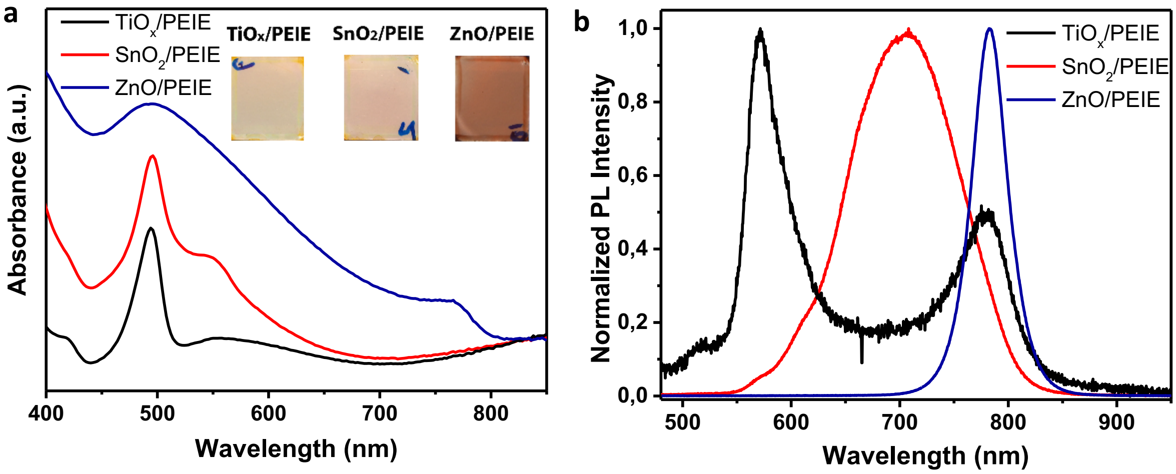

Supplementary Figure 11. Perovskite films (*x*=2.5) with TTDDA on different substrates. a-b, UV-vis absorption (**a**) and normalized PL spectra (**b**) of perovskite films on TiO_x/PEIE, SnO_2/PEIE and ZnO/PEIE substrates, processed from precursors with TTDDA passivation molecule. Insets show images of the thermal-annealed perovskite films on the different substrates.

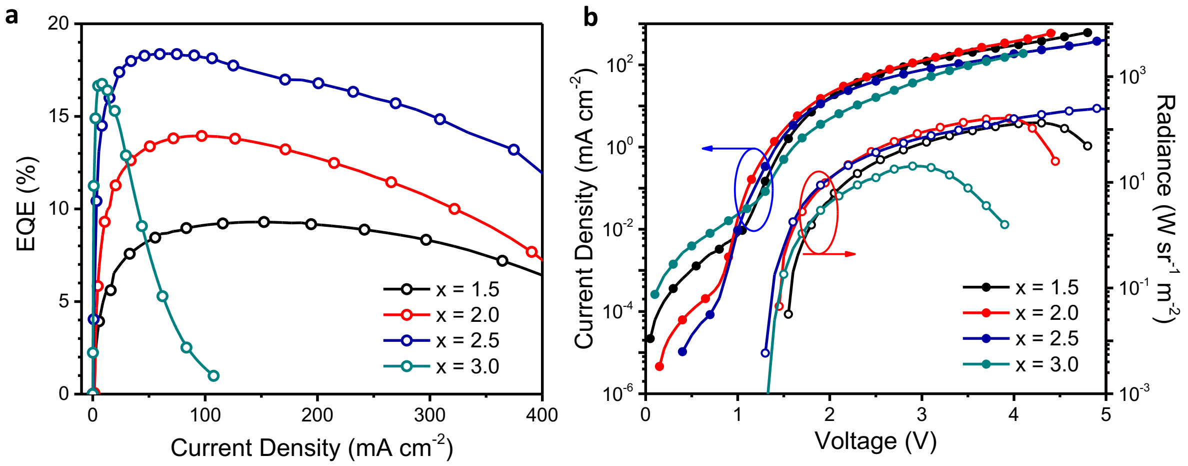

Supplementary Figure 12. Device performance of perovskite LEDs with TTDDA passivation. a-b, EQE-*J* (**a**) and *J* & *R*-*V* (**b**) curves of the perovskite LEDs processed from TTDDA-containing precursors with different *x* values.

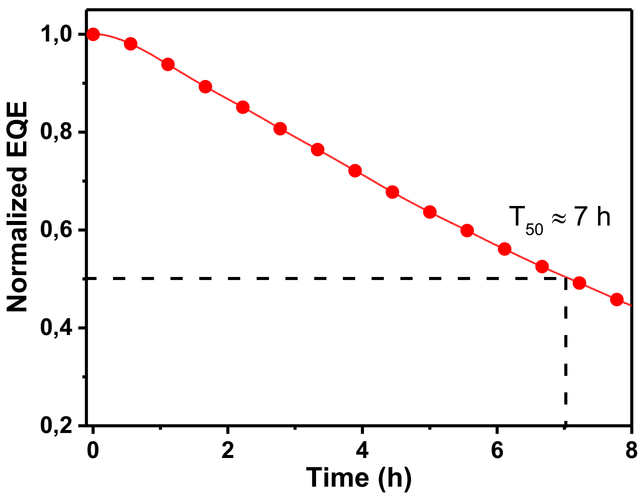

Supplementary Figure 13. Device stability. Operational stability of the optimized perovskite LEDs processed from precursor (*x*=2.5) with TTDDA passivation molecule under a constant current density of 20 mA cm^{-2}.

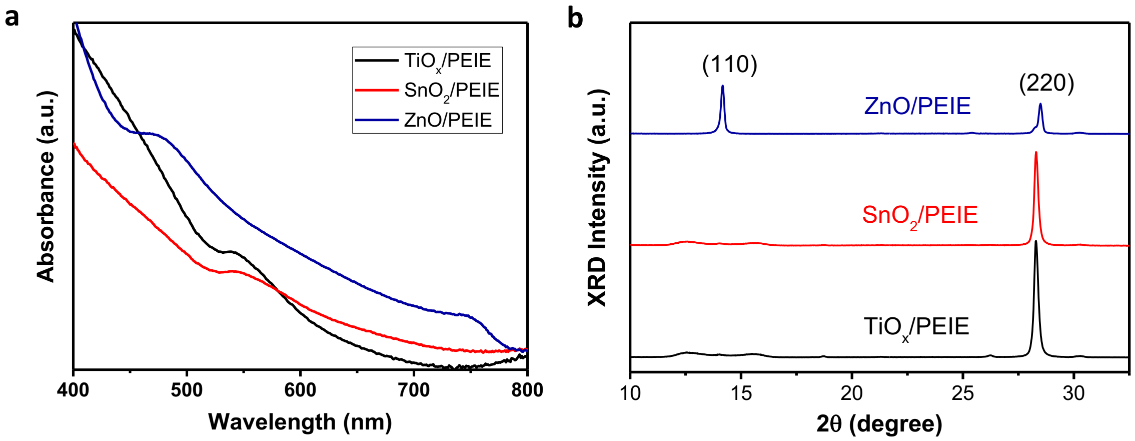

Supplementary Figure 14. Film characterization of MA-based perovskite films. a-b, UV-vis absorption spectra (**a**) and XRD patterns (**b**) of thermal-annealed MA-based perovskite films on different substrates.

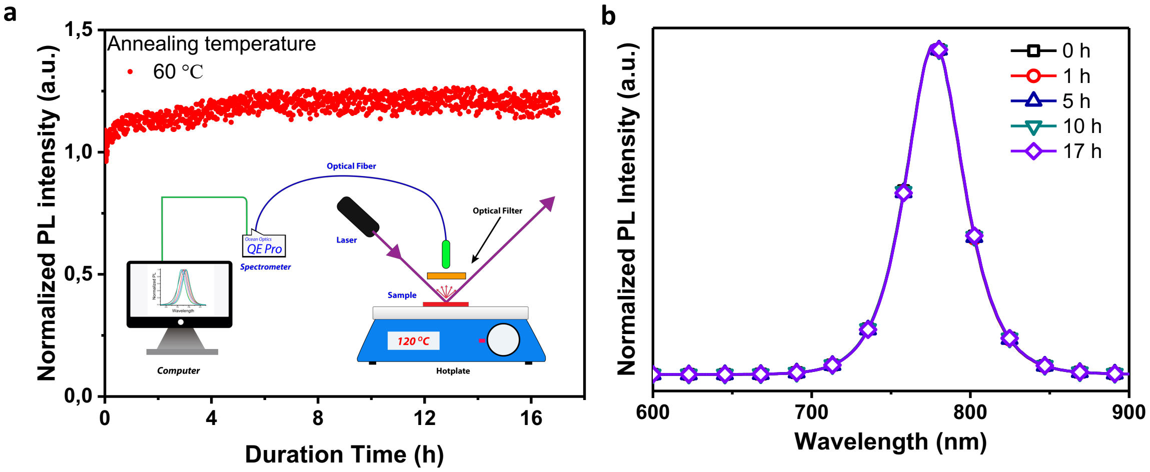

Supplementary Figure 15. Optical stability of perovskite film on ZnO/PEIE. a, PL intensity evolution of perovskite film on ZnO/PEIE at 60 °C as a function of the heating time. Inset shows the schematic of the *in-situ* PL characterization setup. **b**, Normalized PL spectra of the perovskite films during the PL characterization.

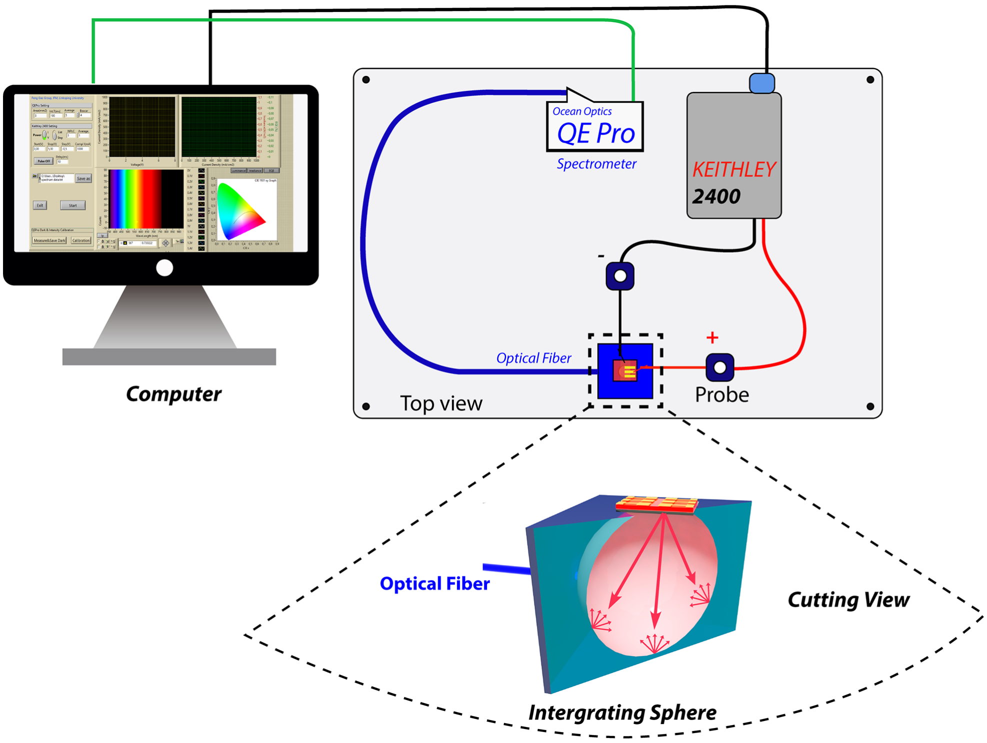

Supplementary Figure 16. Schematic layout of LEDs characterizations setup.

Supplementary Table 1. Device parameters of perovskite LEDs processed from precursors with different component ratios (*x*).

x	EQE (%)	Radiance (W sr^{-1} m^{-1})	EL peak (nm)
**1.5**	4.5 (at 262.8 mA cm^{-2})	90.2 (at 554.2 mA cm^{-2})	783.9
**2.0**	7.1 (at 266.1 mA cm^{-2})	146.5 (at 524.3 mA cm^{-2})	782.5
**2.5**	13.1 (at 193.5 mA cm^{-2})	206.6 (at 369 mA cm^{-2})	778.7
**3.0**	6.4 (at 13.3 mA cm^{-2})	13.7 (at 64.7 mA cm^{-2})	775.7
